# Building Emotional Awareness and Mental Health (BEAM): A Pilot Randomized Controlled Trial of an App-Based Program for Mothers of Toddlers

**DOI:** 10.3389/fpsyt.2022.880972

**Published:** 2022-06-24

**Authors:** Anna L. MacKinnon, Kaeley M. Simpson, Marlee R. Salisbury, Janelle Bobula, Lara Penner-Goeke, Lindsay Berard, Charlie Rioux, Gerald F. Giesbrecht, Ryan Giuliano, Catherine Lebel, Jennifer L. P. Protudjer, Kristin Reynolds, Shannon Sauer-Zavala, Melanie Soderstrom, Lianne M. Tomfohr-Madsen, Leslie E. Roos

**Affiliations:** ^1^Department of Psychology, University of Calgary, Calgary, AB, Canada; ^2^Department of Psychology, University of Manitoba, Winnipeg, MB, Canada; ^3^Department of Psychology, York University, Toronto, ON, Canada; ^4^Department of Pediatrics, University of Calgary, Calgary, AB, Canada; ^5^Department of Radiology, University of Calgary, Calgary, AB, Canada; ^6^Department of Pediatrics and Child Health, University of Manitoba, Winnipeg, MB, Canada; ^7^Department of Psychology, University of Kentucky, Lexington, KY, United States

**Keywords:** digital health (eHealth), maternal mental health, randomized controlled trial, parenting, emotion regulation

## Abstract

**Background:**

Families have faced unprecedented challenges during the COVID-19 pandemic, leading to increased maternal mental health problems and barriers to accessing care. Innovative programs are needed to support both maternal mental health and parenting, and to buffer the long-term impacts of stress on young children. Using a patient-oriented approach, our research team aimed to co-develop and pilot test an App-based psychoeducation and social-connection platform: Building Emotional Awareness and Mental Health (BEAM).

**Methods:**

The co-development process involved a parent advisory board from conceptualization and design, through to direct participation in the program delivery. The BEAM program includes weekly videos and activities based on Unified Protocol therapy modules and emotion-focused parenting strategies, a weekly telehealth group review session, and access to a private online forum for support from other mothers and clinical coaches. A parallel randomized control trial was conducted across two provinces in Canada. Mothers of preschool children (aged 18–36 months old), with moderate-to-severe depression (Patient Health Questionaire-9 ≥ 10), were recruited online and randomized to either the 10-week BEAM intervention or treatment as usual (TAU) control group. Online surveys (ensuring researcher blinding) included questions about feasibility and acceptability of the program and pre/post self-report measures of mental health, parenting, positive coping and child behavior outcomes. The primary outcome measures were symptoms of depression and parenting stress. Data were analyzed using mixed models and an intention-to-treat approach.

**Results:**

65 participants were randomized, by an online allocation tool, to the BEAM (*n* = 33) and TAU (*n* = 32) groups. Engagement was relatively high at the beginning of the program, with 78.8% starting the BEAM App and 70.6% attending ≥1 telehealth session. Most respondents felt socially supported, satisfied with the App, and found it easy to use. Pre-post results indicated interaction effects with greater reductions in overall mental health problems, and specifically anxiety and sleep symptoms, among BEAM vs. control participants. There were also time effects with reductions in depression symptoms across both groups. No significant treatment effects emerged for the other mental health symptoms, parenting problems, positive coping, or child behavior outcomes. Descriptive data are included to highlight possible areas of promise for future large efficacy trials. Technological difficulties and other challenges that may have led to attrition and impacted outcomes are discussed. There were no adverse events related to study participation.

**Conclusions:**

The BEAM program has promise as a novel, feasible and acceptable intervention for improving mental health among mothers of young children.

**Clinical Trial Registration:**

[www.ClinicalTrials.gov], identifier [NCT04772677].

## Introduction

Families have been facing unprecedented challenges during the COVID-19 pandemic, which have led to dramatic increases in maternal mental health problems. A recent meta-analysis indicated that 25–30% of mothers have experienced clinically significant symptoms of depression and anxiety (1). Additional pandemic-related stressors have been identified as key risk factors for families, including isolation, domestic conflict, and a lack of parenting support (2, 3). The absence of standard screening and a backlog at existing services means that the majority of parents will not get access to evidence-based treatments (4, 5). In Canada, only 1 in 10 mothers receive the postpartum mental health care they need (6), resulting in symptom persistence, child mental health problems, developmental impairments and enormous economic impact (7–9). Accessible and scalable programs are urgently needed to build resilience to ongoing stressors and prevent the intergenerational transmission of mental illness to children exposed to maternal mental health problems during the COVID-19 pandemic. Digital mental health interventions have potential for addressing these needs and barriers for families’ care.

Children’s development exhibits high environmental sensitivity in the first 3 years of life, with maternal depression linked to irritable temperament, sleep problems, and socio-emotional impairments later in childhood and adolescence (10–12). Although shared genetic lability may increase children’s likelihood of experiencing mental health problems, environmental factors are understood to moderate this such that risk for long-term mental illness is particularly heightened when children are exposed to persistent maternal depression and other family stressors (13, 14). Social mechanisms through which maternal depression may impact child mental health include low maternal sensitivity (15), maternal modeling of poor emotion coping (16), and negative parenting (e.g., harsh or punitive parenting practices) (17). The parenting stress associated with maternal depression is also established to lead to low-quality interactions and harsh discipline (18), which is particularly concerning in the pandemic context in which children are spending significant time at home.

Although evidence-based treatments exist to address maternal mental illness, there are significant barriers to accessing care, particularly due to the pandemic and its associated public health measures. These include restricted access to mental health clinicians, physical distancing, high costs of individual therapy, closure of existing services, and overwhelming childcare demands (19–21). In our previous research, approximately 20% of mothers with depression during the pandemic reported accessing services (2). Digital interventions provide an accessible, low-cost way to address these barriers and needs for care. Early models of digital mental health interventions show promise for treating adult depression, however dropout rates are high and these programs rarely include parenting-related content and support (22, 23). For example, a meta-analysis of MoodGYM, a web-based program for depression and anxiety, indicated that user completion may be as low as 10% (24). A recent meta-analysis by our team suggested that digital interventions (including videoconferencing, App-based, and Web-based) targeting parent mental health, parenting skills or behavior were associated with improvements in a range of symptoms among parents of children aged 1–5 years old, with effects sizes comparable to in-person interventions (25). Other research highlights the promise of delivering mental health and parenting services through mobile applications with studies revealing App-based intervention programs can improve parent-child interactions (26), mental well-being, sleep, and resilience, and decrease anxiety (27). However, there are few digital programs that target both mental health and parenting skills. One pragmatic RCT of a 4-month nurse moderated App-based program for new mothers found no statistically significant differences compared to care as usual in depression symptoms or parenting problems, despite reporting high engagement and satisfaction (28). While early intervention is critical, the postpartum period has unique challenges and care provisions, thus treatment that extends to early childhood is needed. Addressing intergenerational mental health concerns will require innovative program design methods to simultaneously treat maternal mental illness and address parenting risks—the theorized transmission pathways or causal mechanisms (29).

### Building Emotional Awareness and Mental Health Program Development

The need for scalable online programming became clear early in the COVID-19 pandemic. We and others reported concerningly high levels of maternal mental health problems, which were associated with negative parenting practices (e.g., less responsive parenting, more harsh discipline) (2).

In May 2020, we began consulting with our parent advisory board (10 mothers with lived experience managing depression) by asking the simple question “*What would be helpful for supporting your mental health?*” Their feedback included a request for online support that would allow for connection to other mothers *via* forums or brief (1 h or less) group discussions, contact with therapists, and access to evidenced-based mental health and parenting strategies delivered in short-informational videos. These requests aligned with our knowledge synthesis work highlighting the importance of therapist contact (vs. only didactic materials) to symptom improvement in digital programs (30). Similarly, our previous qualitative research on parenting forums (1,000 posts) during the pandemic highlighted requests for strategies to support family mental health and to feel less isolated (31).

Based on this feedback, and ongoing input from our parent advisory board, we designed the Building Emotional Awareness & Mental Health (BEAM) Program to reduce mental health problems and parenting stress in a scalable, App-based format. BEAM integrates evidence-informed psychoeducation and group therapy with matched emotion-focused parenting skills. A closed-group online forum was also included to promote social support. We chose to use a transdiagnostic treatment approach (i.e., Unified Protocol) to address mental health symptoms characteristic of emotion disorders alongside emotion-focused parenting strategies.

The Unified Protocol is an effective transdiagnostic emotion-focused cognitive-behavioral therapy for emotional disorders in adults (32–34). Unified Protocol targets the underlying processes of aversive/avoidance reactions to emotions and consists of five core modules targeting emotion regulation: mindful emotion awareness; building cognitive flexibility; identifying and preventing patterns of emotion avoidance and emotion-driven behavior; awareness and tolerance of emotion-related physical sensations; and interoceptive and situation-based emotion-focused exposures (35). A recent meta-analysis indicated that Unified Protocol is associated with significant improvement in symptoms of anxiety and depression, in on-site and online formats (36).

Emotion-focused parenting approaches help parents observe and validate children’s emotional reactions, increase communication, and provide support without escalating the situation (37). There are three key processes through which parents help their children learn to regulate emotions: parental reactions to child emotions, talking about emotions, and emotional expressiveness (38). Emotion socialization strategies are demonstrated to be effective for increasing children’s emotional knowledge and reducing challenging emotions (39). This emotion-focused parenting approach was designed to interrupt some of the social mechanisms of parenting risk (e.g., emotional socialization, harsh parenting) through which parent mental health problems are understood to impact child mental health.

BEAM is unique from existing digital programs in that it simultaneously targets both maternal mental health and parenting skills through a therapist-led psychoeducation protocol, combined with a social connection platform. These interrelated skills are designed to reduce maternal mental health problems and synergistically increase supportive parenting behaviors in order to promote family relationships and disrupt the intergenerational transmission of mental illness.

### Current Study

The pilot study had two main objectives: (1) Determine the acceptability and feasibility of version 1.0 of BEAM, and (2) Assess the initial evidence of BEAM on maternal mental health, parenting, and family function outcomes. Although BEAM is designed to target a range of emotion-based parent mental health problems, we chose to recruit based on a single diagnostic criterion (depression) in an effort to have a more homogenous clinical sample. We hypothesized that participation in BEAM would be associated with improvements in mental health and parenting outcomes including reductions in maternal depression symptomatology and parenting stress (primary outcomes) as well as a range of other mental health symptoms (anxiety, sleep problems, anger, alcohol use) and family function (i.e., parenting, coping, child behavior problems; secondary outcomes). The overall aim of this pilot randomized control trial was to use the findings and feedback to inform improvements to BEAM as an accessible and scalable program with potential for rapid, widespread dissemination.

## Materials and Methods

The current investigation represents the second arm of a larger Phase II (preliminary testing) pilot study, following the ORBIT model for developing behavioral treatments (40). See the full study protocol and trial registration on ClinicalTrials.gov (Identifier: NCT04772677). Ethics approval was obtained from the Psychology/Sociology Research Ethics Board (P2020:081) at the University of Manitoba and the Conjoint Health Research Ethics Board (REB20-1933) at the University of Calgary. All study procedures were conducted with the electronic informed consent of participants.

### Trial Design

The current investigation comprised a pilot randomized controlled trial with parallel assignment to the BEAM intervention or treatment as usual (TAU) control group.

Study advertising provided a link to assess eligibility for the trial *via* online screener. Those who met inclusion criteria (listed below) were contacted *via* email with a brief description of the study and to obtain confirmation of their availability for telehealth sessions and time commitment to participate in the trial. Those who confirmed were enrolled in the trial and completed a series of self-report questionnaires before randomization (T1 = pre-intervention assessment). Participants were subsequently randomized to the intervention (BEAM App program) or control (TAU) groups in a parallel 1:1 ratio by computer generated sequencing using an online tool^1^ conducted by a non-affiliate research assistant to conceal allocation. Participants randomized to the BEAM intervention group were asked to register accounts (i.e., create anonymous usernames and passwords) for the BEAM App and to complete their profile in Jane (Jane Software Inc.), a secure online platform used for charting clinical contact. All participants were asked to complete a battery of self-report questionnaires after the 10-week program ended (T2 = post-intervention assessment). Participants randomized to the BEAM App program treatment group were also asked to complete questions assessing feasibility and acceptability at T2.

### Participants

Inclusion criteria were being an adult (aged 18 years or older) mother or other primary caregiver who identify as a woman (e.g., grandmother, aunt) of a child aged 18–36 months old, experiencing moderate to severe depression (Patient Health Questionnaire (PHQ-9) score ≥ 10), living in Alberta or Manitoba, comfortable understanding, speaking and reading English, and available for weekly telehealth sessions (*via* Zoom). Potential participants were excluded if they had significant suicidal ideation, a history of attempted suicide in the past year or self-harm in the past 6 months.

Participants were recruited in the Canadian provinces of Manitoba and Alberta, which both have access to public health care and various social services for families. Online advertisements were used including paid social media posts (e.g., Facebook, Instagram) and postings by community partner agencies *via* electronic mailing lists or public announcements.

*A priori* power was challenging to determine given limited research on eHealth programs for parents of young children and unknown trial aspects such as recruitment feasibility and attrition. Accordingly, we aimed to recruit a sample for one large telehealth group (including breakout rooms) and control group in a set amount of time (May–June 2021). Given participants were being randomized into a group intervention, we were obligated to have a clear start date for the program.

### Intervention

The BEAM program is a novel 10-week App-based digital intervention that combines maternal mental health treatment and parenting skills training with clinician-facilitated peer support and social connection. The primary aim of the program is to improve symptoms of depression and promote a positive parent-child relationship.

**FIGURE 1:**
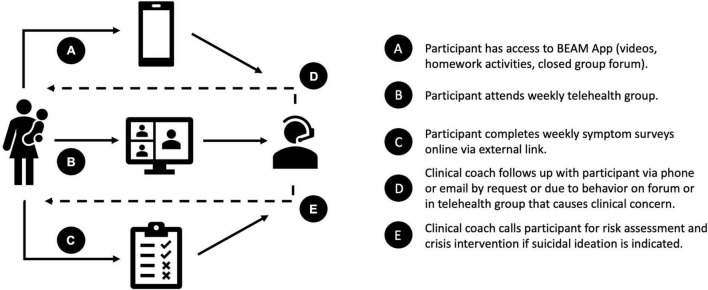
BEAM program components.

There are five main components of the program (see Figure 1): (1) weekly expert-led psychoeducation videos (5–15 min) using (a) adapted Unified Protocol therapy modules (35), which target maternal mental health symptomology, and (b) emotion-focused parenting skills modules (37), which were designed to correspond to the Unified Protocol modules and promote maternal responsivity to children’s emotions (see Appendix Table 1 for weekly module content); (2) a monitored closed group online forum with reflection activities and open discussion to encourage social support; (3) weekly 1-h structured telehealth group sessions (*via* Zoom for Healthcare) to review program content and connect with other participants (41, 42); (4) participants are encouraged to complete weekly activities (i.e., homework) based on the mental health and parenting modules, such as worksheets, reflections, practice exercises and strategies; and (5) participants are also asked to complete a brief weekly survey measuring symptoms of depression and parenting stress. Due to budgetary and technological difficulties (discussed in section “App Interface”), interactive activities and symptom monitoring were not included within the App interface. Therefore, participants were invited to share about activities on the forum and during the telehealth group sessions, and to track their own progress (43) by recording their symptom scores from the weekly survey which was administered externally *via* RedCap link.

A mental health therapist (with a Masters or PhD in clinical psychology, and supervised by a registered clinical psychologist) and parent coach (with lived experience of participating in a similar program) facilitated the forum discussions and telehealth group sessions to increase support, and in turn accountability, consistent with evidence that therapist guidance on eHealth interventions is more effective than self-directed only programs (44). Trial therapists also contacted participants by phone or email if they requested an individual follow-up (e.g., questions about the material, partner or family conflict) or if weekly symptom surveys indicated potential suicidal ideation, for which a risk assessment was conducted and crisis services recommended if necessary. In addition to opportunities for peer support through the forum and telehealth group, mothers were encouraged to identify and engage a person to support their participation in the program (e.g., partner, friend, family member). Research suggests social support is associated with improved adherence and response to psychological interventions (42, 45–47).

The BEAM program was delivered *via* mobile application (accessible by Android and iOS devices). The research team co-developed the evidenced-based content and protocol, while working closely with a digital media company to build the BEAM App. The research team met regularly with the digital media company to determine the App design and function, coordinated extensive user acceptance testing with the parent advisory board for quality assurance, and communicated as needed regarding any necessary updates or repairs.

The control arm of the study was designed to account for the potential effects of time and regular care on change in psychological distress (40). Given ethical considerations to not withhold treatment from mothers experiencing active distress, the TAU group was encouraged to access parenting and mental health resources available in their community. The TAU group was also sent weekly parenting stress and depression symptom surveys to account for change over time; although their scores were displayed on screen immediately following completion, they did not receive instructions to note them or track progress.

All participants in the trial also received an (online) information pamphlet about local parenting and mental health resources (e.g., counseling centers, crisis lines, websites). Data were collected from all participants during the online screener regarding current psychiatric medications and mental health service use in the previous month (e.g., counseling, crisis lines, website).

### Outcome Measures

#### Feasibility and Acceptability

Measures of recruitment, enrollment, and retention were included to assess interest in the overall program and acceptability of run-in procedures.

A narrative description of the different steps and processes involved in developing the digital BEAM program is provided, including considerations and challenges related to building the App interface, hosting platform and data storage, data tracking, and participant communication.

We intended to collect measures of App-based engagement (i.e., log-ins, time spent on app, forum activity, telehealth sessions attended) through Google Analytics and Firebase, but these data were lost due to technical challenges (see “Results” section). In addition, several questions were developed for the pilot study to measure engagement in different components of the program, including videos (“*Have you ever watched a video on the app?*” if yes, “*How many videos did you watch?*”), forum (“*Did you ever participate in the forums?*” if yes, “*How often did you use the forums?*”), and telehealth groups (“*Did you ever participate in a zoom telehealth group?*” if yes, “*How often did you participate in a zoom telehealth group?*”). Intervention group participants were also asked if “*The BEAM program was a good source of social support*” on a 6-point Likert scale from 1 (*Strongly disagree*) to 6 (*Strongly agree*).

The mHealth App Usability Questionnaire (MAUQ) was used to assess participants experience with the BEAM App (48). The MAUQ comprises three subscales that rate ease of use (5 items), interface and satisfaction (7 items), and usefulness (6 items) on a 7-point Likert scale from 1 (*Disagree)* to 7 (*Agree*), where high scores indicate better useability. The subscales demonstrated high internal consistencies in the current sample, with Cronbach α ranging from 0.89 to 0.93.

#### Primary

Change in Maternal Depression was measured using the Patient Health Questionnaire (PHQ-9) (49). This 9-item questionnaire assesses depression presence and severity (mild to severe), where scores ≥10 indicate clinically significant depression and a 5-point reduction represents clinically significant change (50). The PHQ-9 demonstrated good reliability in the current sample, with an internal consistency Cronbach α of 0.68 at T1 and 0.90 at T2.

Change in Parenting Stress was measured using the Parenting Stress Index – Short Form (PSI-SF) (51). This 36-items scale assesses the presence of difficult child behaviors and whether they were stressful for parents, where scores ≥90 indicate clinically significant levels. For the purposes of the current study, a 5-point reduction represents clinically significant change, as this was the approximate mean difference observed for a digital parent training intervention with children aged 2–5 years old (52). The PSI-SF demonstrated high reliability in the current sample, with internal consistency Cronbach α of 0.91 at T1 and 0.92 at T2.

Although we were interested in using the questionnaires as a weekly measure of depression and parenting stress the response rates were very low <20%, so data was not considered informative for the present trial, beyond the need to increase feasibility of mood tracking in future iterations.

#### Secondary

Change in other maternal mental health symptoms was assessed using well-validated self-report measures. Anxiety was measured using the 7-item Generalized Anxiety Disorder scale (GAD-7), where scores ≥10 indicate clinically significant symptoms (53), and a 4 point reduction represents clinically significant change (54). The GAD-7 demonstrated good reliability in the current sample, with an internal consistency Cronbach α of 0.83 at T1 and 0.89 at T2. Anger was measured using the 5-item Patient-Reported Outcomes Measurement Information System (PROMIS) Short Form v1.1-Anger 5a (55), which demonstrated good reliability in the current sample with an internal consistency Cronbach α of 0.80 at T1 and 0.91 at T2. Sleep problems were measured using the PROMIS Sleep Disturbance—Short Form 8a (56), which demonstrated good reliability in the current sample with an internal consistency Cronbach α of 0.84 at T1 and 0.87 at T2. For the PROMIS scales, T-scores of 60.0–69.9 and ≥70 indicate moderate and severe elevated problems, respectively (57), and a 2-6 point reduction in T-scores represents minimal important change (58). Substance use was measured with the 10-item Alcohol Use Disorders Identification Test (AUDIT) (59), which demonstrated good reliability in the current sample with an internal consistency Cronbach α of 0.81 at T1 and 0.83 at T2. For the purposes of the current study, scores ≥6 indicate hazardous use among women and a 5 point reduction represents clinically significant change, as these were the cut-off and approximate mean difference observed for a digital self-help intervention (60).

Change in coping abilities was assessed using the 12-item Self Compassion Scale-Short Form (SCS-SF; Cronbach α of 0.82 at T1 and 0.86 at T2) (61), the 7-item Recent Stressful Events (RSE) hopeful coping scale (Cronbach α of 0.73 at T1 and 0.75 at T2 for the 5 Likert scale items), which was developed based on recommendations from JPB Research Network on Toxic Stress at Harvard’s Center on the Developing Child (62), and the 4-item Couple’s satisfaction index (CSI-4; Cronbach α of 0.95 at T1 and 0.97 at T2) (63).

Change in parenting problems was measured using the 30-item Parenting Scale (PS) which includes lax, overreactive and verbosity discipline styles (64), as well as the 21-item Parenting Young Children (PARYC), which includes setting limits, proactive, and supportive behaviors (65). The PS and PARYC demonstrated good internal consistency in the current sample, with Cronbach α of 0.89 and 0.91 at T1 and 0.86 and 0.86 at T2, respectively.

Change in child internalizing and externalizing behavior problems were assessed by mother report using the 99-item Child Behavior Checklist (CBCL) for ages 1.5–5 years (66). The CBCL demonstrated excellent reliability in the current sample, with an internal consistency Cronbach α of 0.95 at T1 and 0.96 at T2.

### Analytic Approach

All analyses were conducted using SPSS 26.0. Descriptive statistics were computed for the total sample and between groups for demographics and outcome measures at baseline. Longitudinal analysis of covariance, using linear mixed modeling, was conducted in order to test treatment effects on outcome measures. Treatment effects were tested by entering two-way interactions between time and group assignment. An α of 0.05 was used to determine statistical significance. Cohen’s d effect sizes were derived based on recommendations for linear mixed models (67), where 0.20, 0.50, and 0.80 were interpreted as indicating small, medium, and large effects (68). An intent-to-treat (ITT) approach was used, consistent with CONSORT guidelines (i.e., all participants who were randomized to receive enrollment information for the intervention were included in analyses) (69), and missing data was handled using maximum likelihood estimation (70).

Aggregate variables were computed for mental health symptoms (PHQ-9, GAD-7, PROMIS anger and sleep scales, AUDIT), parenting problems (PSI, PS, PAYRC), and positive coping (SCS, CSI, RSE), by converting each measure to a standardized z-score then taking their average. Moderate internal consistency was observed between the standardized scores for mental health symptoms (Cronbach α = 0.68 at T1 and 0.75 at T2), for parenting problems (Cronbach α = 0.60 at T1 and 0.65 at T2), and for positive coping (Cronbach α = 0.50 at T1 and 0.60 at T2).

Exploratory analyses were also conducted to assess clinically significant change in mental health symptoms by creating binary variables for participants in each condition to code whether they achieved the minimum point reduction and/or scored below the clinical cut-offs on the primary and secondary outcome measures at T2. Fisher’s exact tests were used to compare the proportion of participants who exhibited clinically significant change or reached a score below the cut-off.

## Results

### Participant Flow and Recruitment

**FIGURE 2:**
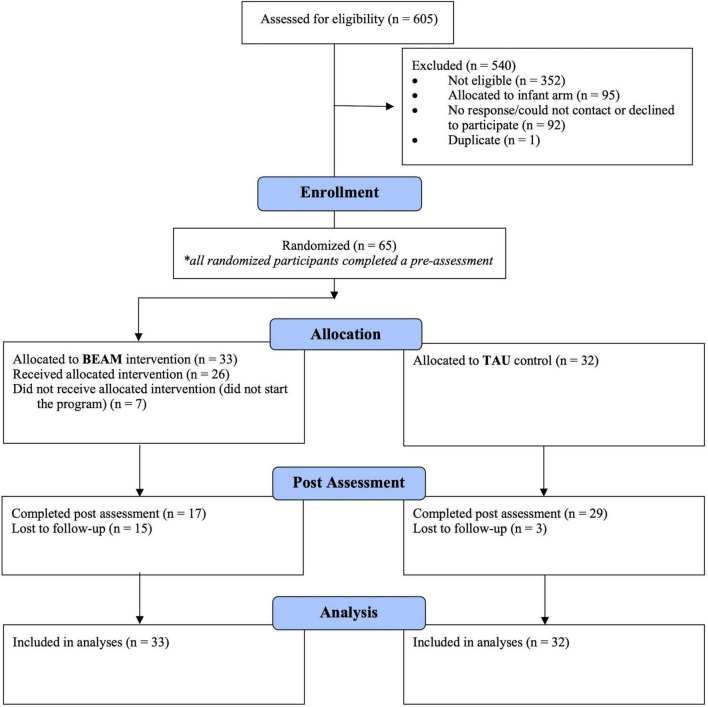
CONSORT flow diagram.

Participant enrolment, allocation, and retention information is provided in the CONSORT flow diagram (Figure 2). Recruitment ran from May 5 to June 16, 2021. Over 600 individuals completed the eligibility screener for both arms of the larger pilot study. Out of the 158 who met inclusion criteria for the preschool arm, 65 (41.1%) were enrolled and completed the T1 assessment, then subsequently randomized to the BEAM program (*n* = 33) or TAU (*n* = 32) groups. Of those participants randomized to the intervention, 78.8% started the BEAM App program (i.e., created a user accounts). The program began on July 5, 2021, with summer break from August 12 to September 8, 2021, and ended on October 13, 2021. T2 post assessment were completed from October 20 to November 22, 2021, and focus groups were conducted from November 9–30, 2022. In terms of attrition, 15 (45.5%) BEAM and 3 (9.4%) TAU participants were lost by the T2 post assessment, a statistically significant difference on Fisher’s exact test (*p* = 0.001).

### Feasibility and Acceptability

Given the significant practical and technical challenges encountered, we first describe these challenges in detail before presenting outcome-related findings. Recommended points of consideration and possible solutions for each of these feasibility and acceptability challenges described below are included in the discussion section (Table 1).

**TABLE 1:** Lessons learned for facilitating the development of a digital mental health program.

Challenges	Recommendations
Participants were lost if they did not send a username and password for the App	Research team creates login information for participants and send to participants
Delayed start date due to technology complications	Ensure technology is in close-to-ready state prior to initiating participant recruitment
Low engagement throughout the summer	Launch in fall or winter
App functionality issues including: Videos not playing on some devices	Research team member available for tech troubleshooting (e.g., re-download App, restart device, check internet connection)
Low attendance at group telehealth sessions	Advertise telehealth groups as mandatory rather than optional, in screener have participants check off times they could commit to attend group sessions
Difficulty finding time across provinces for telehealth sessions	Offer multiple timeslots for sessions
Too many points of contact for participants	Streamline contact with participants and practice the on-ramping process
Participants missing information due to not checking emails	Send critical information and reminders *via* text and email
Engaging participants in the forum in a meaningful way	Have peer coaches facilitate organic discussions within the forum

#### App Interface

Significant challenges arose in our service use agreement with a digital media company to build the BEAM App. This included challenges in communication and expectations regarding feasibly scope in our budget ($120,000) for App development and maintenance. We estimated this amount to be reasonable for developing a pilot therapy program by traditional academic standards, and based on an initial discussion with App-development. However, the features that we believed to be critical to build based on evidence-based best practices and advisory board input likely required a budget of closer to $500,000. As a result, we were not able to include multiple important features including: App-embedded mood tracking, App-embedded group telehealth videos, or any usage-related tailored push-notifications. Instead of dynamic and interactive videos which we hoped to have tech experts co-develop, we created narrated slide presentations to communicate necessary content (see Appendix Table 1 for weekly module content). Although the forum provided a platform for participants to connect with each other and therapists anonymously (by using Bitmoji profile pictures and discrete usernames), the advised forum solution of using third party software was extremely limited because it did not integrate well into the App (e.g., separate window opens), users could not respond directly to comments or tag each other, the threads were difficult to navigate, and is was not visually appealing. We received feedback that small challenges that came up during the pilot trial, such as videos freezing, were not possible to address mid-trial, so we created *ad hoc* solutions such as sending participants YouTube-uploaded videos *via* private channel. The result of these misunderstandings in the App-development costs and process resulted in a limited product with substantial unintended participant burdens due to “friction” from poor ease of use.

#### Hosting Platform and Data Storage

There were lengthy time delays due to legal contract negotiations and service use agreements with the research institutions and digital technology company, in order to align agreements with university intellectual property policies and ensure the processes and product were compliant with local Personal Health Information Act (PHIA) requirements of possible personal health information disclosed on the forums. The digital media company was not able to offer PHIA-compliant server storage, so the university made an exception to standard policy to allow the app to operate within the high-security University protected data storage systems. The time delays associated with the contracting process spanned from September 2020 to March 2021 with the program testing and implementing lasting an additional 3 months, resulting in participants waiting multiple months prior to receiving services (May 2021) and the program running over the summer, which is an undesirable time to test parenting programs due to non-routine schedules. Many participant reported challenges in engaging with material and attending group during the latter half of the summer due to family vacations. After polling the telehealth groups, we made a decision to take a 4-week vacation from early-August to early-September to account for vacations and re-engage in the early fall.

#### Data Tracking

Another challenge was difficulty ensuring relevant usage data (e.g., App logins, time spent on App, activity completion) was being captured on the backend of the App (through Firebase and Google Analytics). Unfortunately, usable data was not collected from Firebase as custom events did not capture our desired variables and data from Google Analytics was lost due to a default *data retention period* setting. This may reflect miscommunications given terminology can differ between researchers and tech developers. Before data collection begins, it is important to determine what variables are needed (70, 71), translate them into *events* that can be tracked, and check whether they are tracked as *automatically collected events* in Google Analytics (e.g., total time spent on the App, session start) or need to be implemented as *custom events* during App development (e.g., time spent on different App screens, tracking when video content is started, paused, resumed, watched completely). This should be done as early as possible during App development as some early decisions can affect the custom events that can be collected (e.g., the choice of video player used in App development determines whether the number of minutes a video is watched can be tracked). In addition, the Google Analytics default data retention setting for storing data should be adjusted to the study period and data should be exported regularly to avoid loss. These settings should be set before study onset as they cannot be adjusted retrospectively, and any data deleted before settings are changed will be unrecoverable. We were able to collect telehealth group session attendance *via* the Jane platform and forum participation data *via* the backend of Vanilla Forums.

#### Participant Communication

Conducting the trial included a significant amount of contact with participants to coordinate enrollment. As the participants were first recruited *via* a public URL leading to our online screener, we emailed eligible participants to confirm their commitment to the program. If participants replied and expressed their commitment, we sent them the link to the online questionnaire and reminders to complete it if needed (i.e., if they had not started or were slow to finish). After randomization, we asked, *via* email, for participants to send us their preferred username, password, contact information, and Bitmoji profile picture for the App and Jane platforms. Many participants required multiple reminders to send us this information. After receiving this information, we manually created accounts for participants across the App, forum, and Jane platforms, and emailed participants with information on downloading and logging into the App. As we registered participants on the app, many required assistance troubleshooting technical difficulties (e.g., login issues, videos not playing) *via* email. During the program, weekly email reminders were sent with links for the symptom surveys (RedCap) and telehealth sessions (Zoom). Check-in emails were sent throughout the program to promote attendance at telehealth sessions.

#### Engagement and Satisfaction

Descriptive statistics for the engagement questions and MAUQ subscales are presented in Table 2. Of the 33 BEAM group participants, 17 (51.5%) completed the measures of feasibility and acceptability. Among these respondents, engagement was relatively high at the beginning of the program with 94.1% self-reporting watching ≥1 video and 70.6% attending ≥1 telehealth session. Video watching amongst respondents ranged from 31.3% reported watching 1–5, 12.5% 6–10, and 31.3% ≥16 of the 31 total videos. Telehealth session participation was also variable, with 25.0% attending 1–3 sessions, 33.3% attending 4–6 sessions, 16.77% attending 7–9 sessions, and 25.0% attending ≥10 sessions. 70.6% of respondents reported participating in the forum, with their use ranging from “*rarely*” to “*once or twice*,” to “*once every 2 weeks.*” Attendance tracking *via* Jane, for the 26 participants who started the BEAM program, indicated that the average number of telehealth sessions was 3.19 (ranging from 0 to 12). Backend data from Vanilla Forums indicated that 14 participants used the forum, with an average of 5.64 posts (ranging from 1 to 15).

**TABLE 2:** Engagement and satisfaction measures for the BEAM App program.

Engagement	*n* (%)^†^	Range
Watched at least 1 video	16 (94.1)	0–16^†^
Participated in forum	12 (70.6)	0–15
Attended at least 1 telehealth session	12 (70.6)	0–10^†^
**MAUQ**	***M* (SD)**	**Range**
Ease of use	24.64 (7.09)	8–35
Interface and satisfaction	33.18 (11.65)	5–49
Usefulness	26.52 (9.38)	6–42

*MAUQ, mHealth App Usability Questionnaire.*

*^†^Out of 17 respondents.*

Respondents indicated that the BEAM program was a good source of social support (58.8% rated ≥4 out of 7 agreement). MAUQ results indicated that the BEAM App had moderate ease of use (e.g., 64.7% rated ≥5 on the “*easy for me to learn*” item), interface and satisfaction (e.g., 56.3% rated ≥5 on the “*Overall, I am satisfied*” item), and usefulness (e.g., 58.8% rated ≥5 on “*useful for my health and well-being*” item).

### Baseline Characteristics

Participant socio-demographic characteristics and outcome measures at baseline are presented for the full sample and by group in Table 3. Mothers were, on average, 33.75 years old, and had 2 children with the toddlers identified for the study being an average of 2.32 years old. Across the full sample, the majority of mothers (81.5%) had completed some form of postsecondary education, most (70.8%) identified as being from European descent, and almost half (46.2%) had an annual household income (before tax) above the Canadian median (approximately $90,000 CAD in 2019). Mean levels of depression, anxiety and anger symptoms, as well as parenting stress, were above the established clinical cut-offs at baseline. After randomization, there were more participants with male children in the TAU group (χ^2^ = 9.600, *p* = 0.002). The imbalance between groups in percentage of participants who reported receiving individual counseling in the month prior to enrolment was not statistically significant (χ^2^ = 2.476, *p* = 0.116).

**TABLE 3:** Sample characteristics at baseline.

		Group	
	Total sample (*N* = 65)	BEAM (*n* = 33)	TAU (*n* = 32)
***n (%)***			
**Socio-demographics**			
European Canadian	46 (70.8)	23 (69.7)	23 (71.9)
Household income > 90K	30 (46.2)	15 (45.5)	15 (46.9)
Post-secondary education	53 (81.5)	26 (78.8)	27 (84.4)
Married	50 (76.9)	27 (81.8)	23 (71.9)
Male child	40 (61.5)	14 (42.2)	26 (81.3)
***M (SD)***			
Age of mother (years)	33.84 (5.34)	33.73 (5.19)	31.90 (5.46)
Age of child (months)	26.02 (6.38)	27.33 (7.26)	24.62 (5.04)
Parity	2.09 (1.09)	2.24 (1.25)	1.94 (0.88)
***n (%)***			
**Treatment history (past month)*^b^***			
Psychiatric medication(s)	27 (41.5)	15 (45.5)	12 (37.5)
Individual counseling	18 (27.7)	5 (29.4)	13 (54.2)
Group counseling	10 (15.4)	4 (25.0)	6 (37.5)
Other services*^a^*	6 (40.0)	2 (23.1)	4 (30.8)
***M (SD)***			
Mental health symptoms*^c^*	0.000 (0.655)	0.014 (0.627)	–0.014 (0.693)
Depression (PHQ-9)	15.98 (4.02)^†^	16.27 (4.27)^†^	15.69 (3.80)^†^
Anxiety (GAD-7)	13.88 (4.68)^†^	14.40 (4.55)^†^	13.34 (4.82)^†^
Anger (PROMIS)	64.90 (6.02)^†^	64.79 (5.16)^†^	65.01 (6.88)^†^
Sleep disturbance (PROMIS)	58.45 (5.88)	58.71 (5.67)	58.18 (6.17)
Alcohol use (AUDIT)	4.09 (4.37)	3.38 (4.03)	4.82 (4.65)
Parenting problems*^c^*	–0.004 (0.736)	0.021 (0.815)	–0.030 (0.656)
Parenting stress (PSI-SF)	93.45 (20.71)^†^	94.00 (22.37)^†^	92.88 (19.19)^†^
Parenting discipline (PS)	3.83 (0.27)	3.82 (0.24)	3.84 (0.31)
Parenting behaviors (PARYC)*^d^*	75.72 (11.08)	75.19 (11.38)	76.27 (10.43)
Positive coping*^c^*	–0.010 (0.729)	0.032 (0.718)	–0.053 (0.750)
Self-compassion (SCS-SF)	2.25 (0.55)	2.34 (0.55)	2.16 (0.55)
Hopefulness (RSE)	12.85 (2.58)	12.97 (2.42)	12.72 (2.77)
Couple satisfaction (CSI-4)	12.91 (5.34)	12.47 (4.88)	13.42 (5.92)
Child behavior (CBCL)	39.35 (23.98)	38.31 (23.13)	40.41 (25.15)

*PHQ-9, Patient Health Questionnaire; GAD-7, Generalized Anxiety Disorder; PROMIS, Patient-Reported Outcomes Measurement Information System; AUDIT, Alcohol Use Disorders Identification Test; PSI-SF, Parenting Stress Index Short Form; PS, Parenting Scale; PARYC, Parenting Young Children; SCS-SF, Self-Compassion Scale Short Form; RSE, Recent Stressful Events; CSI-4, Couples Satisfaction Index; CBCL, Child Behavior Checklist.*

*^a^Other services included: App-based or online mental health programs, instant messaging mental health services, mental health crisis lines, and faith-based counseling.*

*^b^Data collected from online screener.*

*^c^Aggregate of standardized variables.*

*^d^Combined total of subscales.*

*^†^Mean above clinical cut-off.*

There were no statistically significant differences in baseline characteristics between participants who completed the T2 assessment and those lost to attrition, except for more parents of male children among those retained (70.2%) than dropouts (41.2%) according to Fischer’s exact tests (*p* = 0.035). There were also no statistically significant differences in mental health symptoms, parenting problems, positive coping, or children behavior between participants who completed the T2 assessment and those lost to attrition, except that dropouts reported more limit setting on PAYRC subscale (*p* = 0.029).

### Treatment Effects

Results of the mixed model analyses for primary and secondary outcomes are presented in Table 4.^2^ There were no adverse events related to study participation.

**TABLE 4:** Pre-post mixed model results for outcome measures.

	Primary Outcomes		Secondary outcomes			
	Depression	Parenting stress	Mental health	Parenting problems	Positive coping	Child behavior
***Estimate (SE)***						
**Random effects**						
Intercept	12.17*** (3.65)	326.35*** (71.29)	0.301*** (0.070)	0.428*** (0.092)	0.127*** (0.026)	509.42*** (98.84)
**Fixed effects**						
Intercept	15.69*** (0.883)	92.88*** (3.665)	–0.014 (0.117)	–0.040 (0.132)	–0.053 (0.127)	40.41*** (4.27)
Time	–4.33*** (0.927)	–3.72 (2.67)	0.203* (0.096)	0.119 (0.095)	–0.113 (0.093)	1.16 (2.27)
Treatment group	0.585 (1.24)	1.13 (5.15)	0.024 (0.164)	0.078 (0.186)	0.085 (0.178)	–2.10 (5.60)
Time * treatment group	–2.42 (1.45)	–2.35 (4.32)	–0.422** (0.154)	–0.102 (0.155)	0.201 (0.151)	–3.03 (3.77)

*Treatment as usual (TAU) is the reference group. Depression was measured using the PHQ-9, Parenting Stress was measured using the PSI-SF, Mental health was measured using an aggregate (PHQ-9, GAD-7, PROMIS Anger and Sleep Disturbance, AUDIT), Parenting problems was measured using the aggregate (PSI-SF, PS, PARYC), Positive coping was measured using an aggregate (SCS-SF, RSE, CSI), Child behavior was measured using the CBCL.*

*p < 0.05 **p < 0.01 ***p < 0.001.

*Bold values represent statistical significance.*

#### Primary Outcomes

No statistically significant treatment effects (i.e., time*group interaction) were observed for depression or parenting stress, although the effect sizes were moderate (Cohen’s *d* = 0.47 and 0.33, respectively). However, results indicated a statistically significant effect of time for depression, such that symptoms decreased from pre to post assessment across both the BEAM and TAU groups.

For participants who completed T2 differences between groups regarding clinically significant change on primary outcomes was also explored. In terms of depression, 66.7% of respondents from BEAM and 48.3% from the TAU group had clinically significant change (≥5-point reduction) on the PHQ-9. Statistically, Fisher’s exact test indicated this difference was not significant (*p* = 0.176). In terms of parenting stress, 47.1% of BEAM and 48.3% of TAU respondents had clinically significant change (≥5-point reduction) on the PSI-SF, which was not a statistically significant difference on Fisher’s exact test (*p* = 0.590).

#### Secondary Outcomes

Mixed model results for aggregate variables indicated a statistically significant medium treatment effect (i.e., time * group interaction) for mental health symptoms (Cohen’s *d* = 0.71), and moderate but not statistically significant treatment effects for parenting problems nor positive coping (Cohen’s *d* = 0.36 and 0.41, respectively). There was also a moderate but not statistically significant treatment effect for child behavior problems (Cohen’s *d* = 0.41).

Follow-up analyses of the individual scales comprising the mental health symptoms aggregate indicated statistically significant treatment effects (i.e., time * group interaction) for anxiety (*b* = –2.97, *SE* = 1.37, *p* = 0.035) and sleep problems (*b* = –4.43, *SE* = 1.59, *p* = 0.007), where a larger decrease from pre to post assessment was observed among the BEAM participants compared to the TAU group. There were not statistically significant treatment effects for anger (*b* = –0.71, *SE* = 0.81, *p* = 0.387) or alcohol use (*b* = –1.08, *SE* = 0.79, *p* = 0.177).

In terms of anxiety, 52.9% of respondents from BEAM and 34.5% from the TAU group had clinically significant change (≥4-point reduction) on the GAD-7 (Fisher’s exact test; *p* = 0.180). For anger, 64.7% of BEAM and 46.4% of TAU respondents had clinically significant change (≥2-point T-score reduction) (Fisher’s exact test; *p* = 0.189). For sleep, 52.9% of BEAM and 20.7% of TAU respondents had clinically significant change (≥2-point T-score reduction), (Fisher’s exact test; *p* = 0.028). In terms of alcohol use, no respondents from either group had a clinically significant change (≥5-point reduction).

## Discussion

The current pilot randomized controlled trial investigated the development of the Building Emotional Awareness and Mental Health (BEAM) digital program, which simultaneously targeted mental health and parenting skills in mothers of young children. In terms of feasibility and acceptability there was high interest in the digital program at screening, resulting in recruitment of 65 mothers with clinically significant depressive symptoms. Several challenges to building and testing an App-based intervention were identified (see Table 1), which likely impacted engagement, although those who completed the program reported adequate usability and satisfaction. Analyses did not reveal significant treatment effects for symptoms of depression or parenting stress (primary outcomes), however there were greater reductions in overall maternal mental health problems for participants receiving the BEAM program. Specifically, greater reductions in anxiety symptoms and sleep problems (secondary outcomes) were observed among the BEAM vs. TAU participants. No statistically significant effects emerged for parenting problems, positive coping, or child behavior outcomes.

We followed best practices and used a guided approach (with therapists and parent coaches). In terms of acceptability, the MAUQ findings in the current study indicated slightly lower usability than those for a similar App prototype which targeted parent feeding practices with their infants and toddlers (71). Although engagement and attrition in the BEAM program was comparable to other digital interventions that target parent mental health, parenting skills, or child behavior (25), the lower rate of retention in the intervention compared to the control group may suggest program feasibility issues. Indeed, feasibility challenges in the design process and budget available (as is the case with almost any grant-based project) resulted in a non-optimal digital health platform, which may have impacted user experience, engagement, and ultimately mental health. The digital media company emphasized that we should be satisfied with a limited “minimum viable” product for this pilot trial. However, the research team ultimately felt the App was sufficiently compromised so-as to offer a marginally satisfactory test of the promise of materials and therapeutic approach. We tried to overcome the limited functionality of the App on usability and adherence (72), by ensuring learnability of the video content and adding a telehealth group with clinical contact and material review. On the other hand, adding components to make up for App limitations (e.g., external survey weekly link vs. a mood monitoring function, posting about homework on the forum vs. interactive activities within the App) may have inadvertently increased complexity and cognitive demand. Our team is also investigating feasibility and acceptability of the pilot in a separate project through thematic analysis of qualitative data (including open-ended survey questions, forum posts, and focus group interviews), which may provide further insight on helpful components and areas of improvement.

Small to medium treatment effects were observed for mental health symptoms and parenting problems in the current trial, but were not statistically significant due to small sample size and measurement variability. The discrepancy between the linear mixed modeling and clinical significance results for anxiety may reflect the large point reduction required for the GAD-7. Nonetheless, the pattern of change for the BEAM program aligns with meta-analyses indicating larger effects of digital interventions for anxiety than for depression or parenting stress among parents of young children (25). Together this evidence suggests that parental anxiety may be more responsive to digital health interventions, whereas the cyclical nature of depression and related anhedonia may impede improvement and motivation to engage in a treatment with limited clinical contact. The non-ideal summer start of group may also have contributed to depression reductions across groups. Longer-term follow-up will be key in future trials to assess maintenance effects.

Although experiencing parenting stress was not an inclusion criterion for the trial, the mean parenting stress scores of both intervention and control groups was above the clinically significant level. We did not observe any statistically or clinically significant change in parenting stress. The pandemic exacerbated existing gender inequalities with women experiencing more job loss, and greater home and childcare responsibilities (73, 74). Potentially, in the context of chronic pandemic parenting stress, this brief intervention was not enough to decrease it. Future trials with long-term follow up are needed to investigate whether treatment effects may be delayed until acute stressors, particularly related to the pandemic, are resolved. It was also notable that the trial began in the spring of 2021, when pandemic related restrictions were relaxed across Canada. The large reductions in depressive symptoms observed in both groups may have been associated with these easing of restrictions.

Longitudinal studies to examine the sustainability of treatment effects and extent to which reductions in parent mental health problems are linked to changes in parenting and subsequent child mental health will be critical to informing the potential of digital health program to deliver widespread impacts on family mental health. Given that meta-analyses from in-person programs indicate that addressing both mother and child parenting needs leads to ∼50% larger treatment effects, there is a clear need for developing evidence-based digital health programs for parents that prevent child mental illness and its health sequalae in the aftermath of the pandemic.

The acceptability findings and preliminary treatment effects for some of the mental health symptoms suggest that the App-based BEAM program is promising intervention for addressing family mental health and parenting needs during the pandemic and its aftermath. The BEAM App is broadly consistent with priority-setting research in parents of young children which highlights a desire for more support for families to develop healthy coping and emotion regulation (75). Parents have also indicated a need for access to evidence-based information, tailored to their needs, delivered in timely formats (75). Our team is committed to improving the BEAM program and are making multiple systematic improvements to all aspects based on pilot study results and ongoing input from the parent advisory board.

### Strengths and Limitations

The current trial was strengthened by the use of a community-based, participatory action approach to co-develop an accessible, evidence-based digital mental health intervention. The incorporation of peer support and therapist contact directly responds to parents’ needs (75), and follows best practices for digital interventions (42, 44). However, the findings should be interpreted with several limitations in mind. Despite implementing strategies for participant communication (e.g., individualized check-in emails and reminders), retention proved difficult for this digital intervention. Although an intention-to-treat approach was used and dropout analyses indicated no significant differences on outcome measures at baseline, attrition (45.5%) likely reduced power to detect treatment effects. In addition, participants with a more positive perception of the program may have been more likely to complete the engagement and satisfaction measures. Larger efficacy trials are now planned with more participants in order to fully test the intervention in an adequately powered sample. There were no differences between groups in medication or mental health service use at enrollment, however data was not collected on other forms of treatment during the trial, which could potentially obscure the findings. Information on service and resource use will be gathered in future trials. The weekly depression symptom and parenting stress surveys were also sent to the TAU group, and given the benefits of self-monitoring (43, 76, 77), which has been considered as an intervention or perceived mechanism of change (e.g., increasing awareness and reflection) (78–80), this could have functioned as an unintended active control condition. To account for this, our subsequent trials have removed weekly surveys for the comparison groups. Regardless, examining weekly symptom monitoring was not feasible in the current trial due to low completion (<25%), indicating that integration and individualized feedback within the App are needed in future versions of the program. Lastly, the generalizability of the results is restricted by the demographics of the sample and should be replicated among parents with more diverse socioeconomic backgrounds, among equity seeking groups, as well as non-female identifying caregivers.

### Future Directions

Investing in maternal mental health early, before problems are entrenched, is expected to yield high health and economic benefits by preventing the long-term consequences of maternal depression from becoming embedded in children’s biological and behavioral development (81). Although there were no prespecified criteria for proceeding to a definitive trial given the novelty of the field, our team is using a rapid cycle iteration approach, following the IDEAS (Innovate, Design, Evaluate, Adapt, Scale) Impact Framework™ from the Harvard Center on the Developing Child, to improve our ability to affect change in the BEAM program outcomes based on feedback from focus groups with participants. These improvements include simplified content delivery, streamlined communication with participants, greater synergy between mental health and parenting content, allowing parent coaches to fully facilitate the forums, and improving the user experience in the App. Indeed, we have secured further funding to update the App and program content and will be launching several larger trials that incorporate more peer-coaching, and involvement from service providers at local agencies holding tailored expertise to community needs. Version 1.2 of the BEAM program is being tested in a larger Phase III (efficacy) randomized controlled trial and a pragmatic trial with a community organization, as well as plans for a full App re-build in an embedded longitudinal cohort study for mothers who were pregnant during the pandemic.

## Conclusion

Results highlight both feasibility limitations in version 1.0 of the BEAM program alongside significant promise to improve family mental health through an App-based digital intervention.

## Data Availability Statement

The raw data supporting the conclusions of this article will be made available by the authors, without undue reservation.

## Ethics Statement

The studies involving human participants were reviewed and approved by Psychology/Sociology Research Ethics Board (P2020:081) at the University of Manitoba & the Conjoint Health Research Ethics Board (REB20-1933) at the University of Calgary. The patients/participants provided their written informed consent to participate in this study.

## Author Contributions

LR, LT-M, KR, RG, MS, JP, and GG contributed to the conceptualization and design of the study, as well as funding applications. SS-Z was consulted regarding adaptation of the Unified Protocol treatment program. KS, MRS, JB, and LP-G contributed to the trial coordination and data collection. AM and LB were clinical trial therapists. AM and KS conducted the data analyses and synthesis. AM, KS, MRS, CR, LP-G, LT-M, and LR contributed to the preparation of the manuscript for publication. All authors have reviewed and approved the final version.

## Conflict of Interest

The authors declare that the research was conducted in the absence of any commercial or financial relationships that could be construed as a potential conflict of interest.

## Publisher’s Note

All claims expressed in this article are solely those of the authors and do not necessarily represent those of their affiliated organizations, or those of the publisher, the editors and the reviewers. Any product that may be evaluated in this article, or claim that may be made by its manufacturer, is not guaranteed or endorsed by the publisher.

## Footnotes

1
https://www.randomlists.com/team-generator

2 Including child sex as a covariate did not change the pattern of findings for any of the mixed model analyses. Therefore, the results are presented here without covariates.

## Appendix

**TABLE A1:** BEAM weekly module topics.

Week	Module	Mental Health	Parenting
1	This stuff is tough	Introduction to the UP program Checking in with your mental health (self-monitoring)	Debunking parenting myths Finding your personal values
2	How do i get motivated?	Setting (SMART) goals Maintaining motivation Decisional balance exercise	Using mindfulness and self-compassion to identify moments of joy, feel less stressed, and improve relationships with children
3	The payoff to understanding emotions	The functions of emotions Three component model of an emotional response (cognitive, physiological, behavioral)	Teaching children about emotions through play and stories
4	How do my emotions work?	Putting emotions into context by identifying antecedents and consequences	Parents as co-regulators; responding with awareness and moving on together
5	Appreciating the rainbow amidst the storm	Developing and practicing mindful emotion awareness and self-compassion Meditation exercises	Loving your whole child (both positive and challenging qualities) Preparing ahead for difficult situations Responding with compassion
6	Getting unstuck	Identifying and changing negative thinking patterns and beliefs with cognitive flexibility	Motivating flexibility and adapting to children’s needs to help navigate tough situations (transition toolbox)
7	Finding a better path when emotions are high	Identifying and countering emotional behaviors with alternative actions	Using alternative actions to help children manage emotional reactions
8	Feeling our feelings	Understanding physical sensations Physical exposure exercises	Coping strategies to regulate physical sensations while parenting
9	Exploring Your options	Developing an emotional exposure hierarchy (situational and imaginal)	Skills and techniques for managing tantrums
10	Where do i go from here?	Review of skills and progress Developing a practice plan	Identifying what you need to feel well and balanced
